# Equipping Pacific emergency medical teams for self-sufficient health emergency response in remote and resource-limited island settings

**DOI:** 10.5365/wpsar.2023.14.6.1032

**Published:** 2025-06-02

**Authors:** Pierre-Yves Beauchemin, Erin E Noste, Jan-Erik Larsen, Sean T Casey

**Affiliations:** a World Health Organization Regional Office for the Western Pacific, Suva, Fiji.; b Department of Emergency Medicine, University of California San Diego, San Diego, California, United States of America.; c World Health Organization Regional Office for the Western Pacific, Manila, Philippines.; d School of Population Health, University of New South Wales, Sydney, New South Wales, Australia.

## Abstract

**Problem:**

Pacific island countries and areas represent some of the most disaster-vulnerable locations in the world, facing a range of natural and infectious hazards along with incredibly challenging logistics and limited human resource pools.

**Context:**

The World Health Organization supports the development of emergency medical teams across the Western Pacific Region. Since 2021, one aspect of this support has been supplying health emergency response equipment called cache kits for these unique island contexts. This report describes the process of designing and implementing standardized cache kits for these teams.

**Action:**

Emergency medical team cache kits were designed and sourced using a semi-structured six-step approach: 1) problem identification and review of existing literature; 2) targeted key informant interviews and stakeholder consultations; 3) the alignment of cache with the goals and objectives of the teams’ operations; 4) creation of the kits; 5) local and international procurement of selected items; and 6) monitoring of the delivery of cache to destination countries.

**Outcome:**

The Organization procured specialized cache kits for 12 teams across the Pacific subregion. They comprise portable, durable, lightweight equipment that enables teams to deliver high-quality emergency medical care in remote and resource-limited island contexts.

**Discussion:**

The Organization's centralized procurement of the cache kits in the Pacific aimed to facilitate nationally led health emergency responses, enhance team interoperability in the subregion, and ensure access to high-quality equipment in resource-constrained locations. The model established in the Pacific could serve as a blueprint for national emergency medical teams in low- and middle-income countries globally.

## PROBLEM

Pacific island countries and areas (PICs) represent some of the most logistically challenging locations, with thousands of remote islands spread over millions of square kilometres (km^2^) of ocean territory. For instance, Federated States of Micronesia (FSM) encompasses 607 islands dispersed over an extensive maritime area of nearly 3 million km^2^, comparable in size to India, despite its land area being only 702 km^2^ (or 0.02%). (*1*)

Earthquakes, landslides, volcanic eruptions, storm surges, floods, cyclones/hurricanes/typhoons and tsunamis cause extensive damage, lead to injuries and deaths, aggravate the risks and intensity of diseases, and negatively impact access to health-care facilities. (*2*) While some PICs have areas of high population density, many comprise dozens or hundreds of islands spread over massive distances, with the outer islands often contending with a lack of physical infrastructure, limited transport connections, and suboptimal communication and power networks. Routine logistical challenges are frequently compounded in disasters, causing system disruptions and an interactivity of deleterious factors, accelerating the rate at which a disaster may escalate. (*3*) Infrastructure damage, reduced transport connections, communication and power network disruptions, physical damage to health-care facilities and population displacements are only a few examples making response operations complex and presenting a range of challenges in the provision of timely medical assistance to affected populations. With improvements in the understanding of disaster relief, emergency logistics has become a key aspect in increasing the efficiency of relief and the alleviation of disaster impacts. (*3*)

## CONTEXT

The World Health Organization (WHO) Emergency Medical Team (EMT) Initiative, launched in 2010 following the Haiti earthquake, aims to enhance the development and deployment of medical teams to respond to public health emergencies and disasters. (*4*) EMTs consist of trained local health professionals such as doctors, nurses and paramedics who can deploy and provide essential medical care in the immediate aftermath of a crisis, supported by logisticians and emergency management professionals. (*4*)

**Fig. 1:**
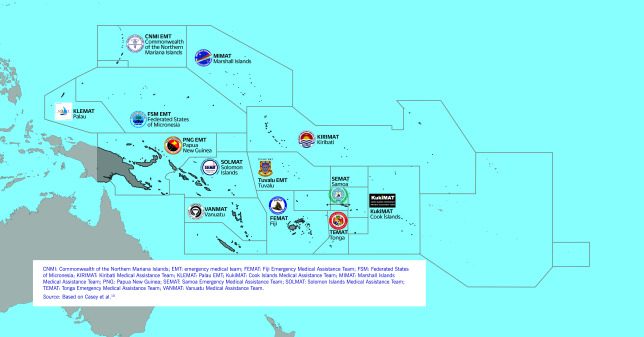
Pacific island countries and areas receiving tailored emergency medical teams cache kits, 2021–2022

Since 2017, WHO and its partners have supported the development of EMTs across 13 PICs. These include one internationally classified EMT, the Fiji Emergency Medical Assistance Team (FEMAT), as well as 12 national EMTs in Cook Islands, FSM, Kiribati, the Marshall Islands, the Commonwealth of the Northern Mariana Islands, Palau, Papua New Guinea, Samoa, Solomon Islands, Tonga, Tuvalu and Vanuatu (**Fig. 1**). (*5*-*11*) In January 2021, the WHO Regional Office for the Western Pacific embarked on a research and consultation process with experts to determine the optimal equipment and supplies (known as the “EMT cache”) for EMTs in the Western Pacific Region. This initiative was guided by the *Classification and minimum standards for emergency medical teams* (also referred to as the Blue Book), and took into account the unique operational context in the Region. (*4*) Cache kits are pre-assembled and pre-packed (commonly referred to as “kitted”) by teams in-country, and stored locally in backpacks and cargo boxes for rapid identification, pickup and deployment. These kits are prepared according to a team's scope of work and expected interventions in the field. Through this process, the Regional Office designed kits, and procured and delivered supplies to EMTs across the Pacific. (*4*, *9*-*12*)

[**insert** Figure 1]

This paper highlights actions taken to support EMTs in small island/large ocean states in the Pacific to enhance their health emergency response capabilities by identifying and sourcing appropriate equipment to enable teams to deploy swiftly and provide quality medical care to affected populations.

## ACTION

The design, development and procurement of a standardized EMT cache kit for Pacific EMTs followed a semi-structured six-step approach.

### Step 1: Problem identification and literature review

Before developing a tailored cache kit for Pacific EMTs, no standardized equipment list existed for national teams. The Regional Office launched the process by identifying key deployment challenges, analysing after-action reviews (AARs) from past responses and assessing international EMT standards. A literature review of EMT disaster deployments since the 2010 Haiti earthquake was conducted to further refine field equipment needs. (*9*, *13*, *14*)

### Step 2: Targeted expert interviews and stakeholder consultation

Semi-structured key informant discussions were held with logistics, medical, and water, sanitation and hygiene (WASH) experts with relevant Pacific EMT deployment experience. These discussions explored past challenges, the use of specific equipment in remote island settings and lessons from previous responses. Consent was obtained to use insights from these interviews for this article. Experts from existing EMTs were consulted, including the Australian Medical Assistance Team (AUSMAT), the Fiji Emergency Medical Assistance Team (FEMAT), the New Zealand Medical Assistance Team (NZMAT), the Solomon Islands Medical Assistance Team (SOLMAT), the Vanuatu Medical Assistance Team (VANMAT), Team Rubicon based in United States of America and other subject matter experts throughout the EMT global network. The process considered the numerous EMT operational support requirements, including medical services, staff living quarters, site power, radio and satellite communications, food preparation, safety and security, and WASH for both the team and patients in the health-care setting.

### Step 3: Goals and objectives of EMT cache kit

Following the outlined steps, the Regional Office defined the EMT cache kit’s goal: a self-sufficient, fully equipped kit enabling teams to deliver high-quality emergency and clinical care for at least 3 days with minimal or no footprint on local resources.

### Step 4: EMT cache kit list creation

Understanding the lifecycle of emergency deployment was key to developing Pacific-tailored EMT cache kits, focusing on surveillance, on-the-ground tasking, monitoring, demobilization and preparation for re-tasking. To streamline this process, EMT activities were grouped into three main categories: logistics support, medical care delivery and WASH management, each covering key aspects of crisis response. Defining these categories helped structure the organization of supplies and equipment within the cache kits, frequently relying on Sphere guidelines. (*13*) A systematic approach ensured each category was further divided into subcategories, detailing specific activities, supplies and required quantities for EMT deployments.

### Step 5: EMT cache kit procurement

Supplies were researched based on strict specifications to ensure durability and sustainability. Suppliers were vetted through a global call for offers, requiring proof that all items met durability, sustainability and minimum quality standards.

### Step 6: Monitoring cache delivery and EMT feedback

A dedicated tracking system was developed to monitor and record each shipment and the delivery status of the cache items across the Region.

## OUTCOMES

This section outlines the key challenges, solutions, and their impact on strengthening EMT response capabilities in the Pacific for each of the above steps.

### Step 1: Problem identification and literature review

Understanding the challenges of disaster deployments in the Pacific was the first step in shaping a relevant equipment and supply list for national EMTs. Key insights from AARs informed the development and procurement of EMT cache kits, highlighting the following themes:

sea transport is difficult to organize and coordinate, and is often the *de facto* option for reaching remote islands;
navigating open water on small vessels is dangerous;
delays occur due to a lack of pre-planning and pre-packaging of EMT (kitted) cache;
securing temporary accommodation for deployed teams in remote areas can be difficult;
accessing fresh and nutritious food for the team not only burdens afflicted local markets further but also proves challenging in devastated areas;
communication failures between deployed teams and the Health Emergency Operating Centre occur due to the absence of adequate, functional equipment, such as very high frequency radios or basic satellite communication devices;
ensuring fuel and power availability is a persistent issue;
high humidity and salt exposure accelerate wear and tear, causing medical devices, radios and power systems to fail during deployment;
mosquitoes and other vectors thrive in tropical environments, rapidly increasing malaria, dengue and leptospirosis risks for both teams and patients; and
medical waste disposal is challenging on remote islands, creating biohazard risks and contamination if not properly managed.

Published literature on EMT logistics is scarce. At present, no common or baseline standard kit exists for EMTs, either globally or in specific contexts, such as tropical islands or extreme cold conditions. During the cache kit development process, the Blue Book’s international EMT standards were a key reference, focusing on Type 1 Mobile (T1M) teams – the lightest and most agile EMT configuration – due to their adaptability for remote deployments.

### Step 2: Targeted expert interviews and stakeholder consultation

Expert discussions underscored the critical need for timely supply delivery, highlighting procurement and transport as major challenges following a sudden-onset disaster. In the Pacific, transitioning from a fixed to a mobile operational model proved the most effective strategy. Overcoming transport obstacles requires mapping in advance of all available resources, including military, law enforcement and private assets such as fishing boats. Experts also emphasized that T1M EMTs should not be confined to a single site – they should be able to cover multiple villages or islands, especially after floods or storms when communities are displaced, and health facilities are inoperable.

### Step 3: Goals and objectives of EMT cache kit

The EMT cache kit was designed to enable national EMTs in PICs to deploy rapidly with minimal logistical burden. Research and planning ensured the kit remained light, mobile and optimized for small aircraft or sea vessel transport to reach remote and vulnerable populations. The WHO team built the cache kit around several key design principles:

the cache is built to meet T1M EMT requirements, ensuring teams have the necessary tools for rapid deployment;
all equipment and supplies adhere to EMT Blue Book standards but are tailored for national, rather than international, deployments;
medical consumables and pharmaceuticals are excluded, requiring national EMTs to source their own supplies based on country-specific needs;
each kit supports a fully autonomous 3-day deployment without reliance on external resupply;
the setup is designed for a four- to six-member team, including one doctor, three nurses, one team leader and one logistician;
all supplies are built for durability, capable of withstanding multiple deployments in high humidity and saltwater conditions; and
weight and size are optimized for transport by small aircraft and sea vessels with limited cargo capacity.

### Step 4: EMT cache kit list creation

The curated Pacific EMT cache kits feature portable, durable and light equipment, built to withstand several deployments before requiring significant maintenance or replacement. A detailed breakdown of the EMT cache specifications for a four-member team (typically one doctor, two nurses and one logistician) is provided in **Table 1**. Excluding pharmaceuticals, medical consumables and food, a cache kit contains 135 items, weighs approximately half a tonne and is fully scalable and modular, meaning cache kits can be aggregated if a response requires scaling up. It is divided into three categories of supplies: mobile clinic, logistics and operations, and WASH. Of the total, 58% fall under logistics and operations, 23% under medical and 19% under WASH – underscoring the practical realities and self-sufficiency required for remote island deployments.

**Table 1:** Illustrative contents of an emergency medical team cache kit to support a four-member team for  3 days of self-sufficiency

Category	Subcategory	Item	Quantity
**Medical**	**Mobile clinic**	**Medical backpacks**	**3**
		**Tent, 16 m2**	**1**
		**Foldable table**	**2**
		**Foldable chair**	**2**
		**Foldable, portable stretcher made of water-resistant, nonwoven material**	**3**
		**Foldable patient cot**	**4**
		**Tarpaulin, 4 m x 5 m (privacy screen)**	**2**
	**Medical equipment**	**Narcotic lockbox**	**1**
		**Slap-on triage bands (multiple colours)**	**50**
		**Vaccine carrier**	**2**
		**Tourniquet**	**10**
		**Blood pressure cuff (adult)**	**4**
		**Blood pressure cuff (paediatric)**	**4**
		**Pulse oximeter (adult)**	**4**
		**Pulse oximeter (paediatric)**	**4**
		**Otoscope**	**1**
		**Ophthalmoscope**	**1**
		**Spare batteries for otoscope-ophthalmoscope**	**8**
		**POC glucometer**	**2**
		**Glucometer test strips (pack of 50)**	**4**
		**Safety lancets (box of 200)**	**2**
		**POC haemoglobin machine**	**1**
		**POC haemoglobin testing strips (pack of 50)**	**2**
		**Timer (for laboratory management)**	**2**
		**Hand-held ultrasound machine**	**1**
		**Ultrasound gel, 250 mL**	**2**
		**Autoclave**	**1**
		**Automatic external defibrillator**	**1**
		**Trauma shears**	**8**
		**Sharps container**	**4**
		**Plastic cups for patient use (pack of 100)**	**1**
**Logistics**	**Personal ** **deployment kit** **Base camp**	**Backpack, 75 L, with rain cover**	**4**
		**Dry bag, 35 L, for large items such as clothing**	**4**
		**Dry bag, 5 L, for personal effects such as phone or wallet**	**4**
		**Rain poncho**	**4**
		**Inflatable sleeping pad**	**4**
		**Inflatable camping pillow**	**4**
		**Sleeping bag or sleeping sheet adapted to local temperatures**	**4**
		**Lightweight travel camping sheet**	**4**
		**USB rechargeable headlamp**	**4**
		**Camping personal towel (pack towel)**	**4**
		**Water filter bottle**	**4**
		**Hand sanitizer 70%, min 200 mL**	**4**
		**Ultralight folding chair**	**4**
		**Knife with can opener**	**4**
		**Waterproof solar power bank**	**4**
		**Mess kit with mug and spork**	**4**
		**Waterproof notepad with pen**	**4**
		**Ultralight, water-resistent personal medical kit**	**4**
		**High-visibility vest**	**4**
		**Clear safety glasses**	**4**
		**Safety whistle**	**4**
		**Earplugs (pair)**	**8**
		**Safety work gloves (pair)**	**4**
		**Zipper-closure plastic bag (for toilet paper)**	**4**
		**Sunblock, SPF 50**	**4**
		**Personal flotation device, up to 95 kg**	**4**
		**EMT sun hat**	**4**
		**EMT t-shirt and trousers (set)**	**16**
		**Small ABC fire extinguisher**	**1**
		**Tent, 16 m2**	**1**
		**Waterproof tent, 42 m2 (voluminous and heavy; its deployment is pending on the severity of the situation and available transportation options)**	**1**
		**Fence stake (camp perimeter and patient flow), pack of 50**	**1**
		**Parachute cord roll, 4-mm outdoor binding rope, length 100 m (camp perimeter and patient flow)**	**2**
		**Machete**	**1**
		**Axe**	**1**
		**Folding saw (wood)**	**1**
		**Extra-strong steel tent peg, minimum length 25 cm**	**20**
		**Car DC-to-AC inverter, minimum 500 W**	**1**
		**Universal plug adaptor with USB**	**3**
		**Outdoor extension cord, minimum length 6 m**	**2**
		**Power strip, surge protector with 6 universal outlets**	**2**
		**Gasoline 1 kVA generator/inverter**	**2**
		**International Air Transport Association-compliant fuel tank, 20 L**	**4**
		**Foldable shovel**	**1**
		**200-piece toolbox**	**1**
		**Heavy duty zip ties (pack of 200)**	**2**
	**Base camp**	**Portable lamp with cord and hang-up hook**	**2**
		**Solar light**	**2**
		**Space light bulb, 100 W**	**2**
		**Two-person tent**	**4**
		**Reflective guyline tent rope, diameter 3–5 mm, ** **length 15 m**	**15**
		**Portable shower and toilet pop-up tent**	**4**
		**Team first aid kit**	**1**
		**Duct tape, large**	**4**
		**Water-resistant fabric patch (tent, mattress, ** **pillow repair)**	**1**
		**Heavy-duty, water-resistant case, 60 L**	**8**
	**Kitchen**	**Multifuel compact stove**	**2**
		**Outdoor cooking set**	**1**
		**Waterproof matches, 100-pcs pack**	**2**
		**Zipper-closure plastic bag (for food storage)**	**1**
		**Magnesium flint fire starter**	**1**
		**Ultralight kitchen set**	**1**
		**Cleaning scrub sponge**	**1**
		**Biodegradable soap, liquid**	**1**
		**Regular garbage bag, roll**	**1**
		**Fishing rod and a few accessories**	**1**
	**Communications**	**Hand-held, portable satellite communicator enabling two-way messaging and emergency location sharing**	**1**
		**Portable satellite internet router (pending available coverage)**	**1**
		**Two-way VHF radio**	**4**
	**Office**	**Laptop**	**1**
		**Monochrome laser printer**	**1**
		**Toner replacement**	**1**
		**Watt voltage transformer converter with built-in regulator**	**1**
		**Pen**	**8**
		**Printing paper (pack of 500 sheets)**	**1**
		**Water-resistant accordion file organizer**	**4**
		**Letter-size clipboard**	**4**
		**Plastic sheet protector (pack of 50)**	**1**
**WASH**	**Patient washroom/sanitation equipment**	**Telescoping tent poles**	**12**
		**Adapted bucket as a toilet**	**4**
		**Tarpaulin, 2 m x 6 m, opaque**	**2**
		**Biohazard waste disposal bags (roll of 30)**	**2**
		**Human waste treatment powder**	**1**
		**Handwashing station, ultralight, foot pump ** **(adapted bucket)**	**4**
	**Patient washroom/sanitation equipment**	**Disinfecting wipes, minimum 75 pcs**	**6**
		**Toilet paper**	**8**
		**Regular soap bar**	**8**
	**Temporary morge**	**Peg, 30 cm (to anchor tarpaulin)**	**4**
		**Tarpaulin, 4 m x 5 m**	**2**
		**Body bag (adult)**	**5**
	**Vector control**	**Bed net**	**6**
		**Insect repellent**	**6**
	**Water storage**	**Collapsible water container, 20 L**	**20**
		**Bucket with lid, 20 L**	**10**
	**Water testing**	**Free residual chlorine test strips, 50 pcs**	**2**
		**Turbidity test device**	**1**
		**pH level test device**	**1**
	**Water treatment**	**Hand-operated desalination unit, 4.5 L/hr**	**1**
		**Potable aqua chlorine dioxide water purification tablets, 67 mg, minimum 20 pcs per pack**	**6**
		**Gravity-fed filtration system, 4 L**	**10**
		**Team water filtration system (potable), 50 L**	**1**
		**Heavy-duty chemical-resistant gloves**	**1**
		**Hand-held electrochlorinator**	**1**
		**Multipurpose bleach, 4 L**	**1**

cm: centimetre; EMT: emergency medical team; kg: kilogram; kVA: kilovolt-ampere; L: litre; m: metre; m^2^: square metre; mg: milligram; mL: millilitre; mm: millimetre; pcs: pieces; POC: point-of-care; VHF: very high frequency; W: watt; WASH: water, sanitation and hygiene.

The mobile clinic category includes a light tent and packable tables, chairs, stretchers and patient cots, along with essential medical equipment such as a point-of-care glucometer, ultrasound machine, autoclave and automatic external defibrillator. The logistics and operations category includes a personal deployment kit for each team member, along with base camp supplies for daily living needs, a camp kitchen, communication devices and a mobile office to coordinate operations in the field. The WASH category ensures sanitation, hygiene and water safety, providing washroom facilities, vector control, a temporary morgue, and equipment for water testing, treatment, control and storage. In some remote island locations with limited freshwater sources, a hand-operated desalination unit was included in the cache. (*13*)

### Step 5: EMT cache kit procurement

The Regional Office procured cache kits for 12 EMTs across the Pacific in 2021–2022, adopting a bulk procurement strategy after consultations with Pacific partners. Global suppliers were contracted to source and deliver the equipment directly to each country to streamline logistics and reduce costs.

### Step 6: Monitoring cache delivery and EMT feedback

A cargo tracking system fed a dashboard visualizing the deployments' progress by country, indicating the estimated arrival times, shipping references and volume measurements of each shipment. This allowed the teams to coordinate customs clearance and secure proper storage upon arrival. WHO continues to evaluate EMT feedback and best practices, focusing on equipment effectiveness and cache readiness to refine future deployments.

## Discussion

With an improved understanding of disaster relief, emergency logistics has become a key component in improving relief efficiency and alleviating disaster impacts. (*14*, *15*) Despite this progress, PICs often face severe gaps in equipment availability, supply chain resilience and operational readiness. The EMT cache kit initiative addressed these challenges by providing standardized, quality medical equipment to national response teams and ensured better preparedness in resource-limited settings. Beyond improving access, this approach also strengthened regional interoperability, an objective aligned with the EMT 2030 Strategy. (*16*) Effective implementation is ensured by the participation of Pacific EMTs in dedicated training sessions and hands-on exercises, enabling them to develop operational familiarity and practice using their new equipment in real-world conditions. (*17*, *18*)

Although the process strengthened cache availability for national EMTs in the Pacific, it also revealed several important limitations. First, while the cache kit design drew heavily on key informant interviews with EMT logisticians and clinicians who had direct deployment experience across the Pacific, the study did not include structured field testing or follow-up user evaluations post-deployment. As such, the long-term appropriateness of the kitting approach, as experienced by teams after delivery, remains an area for further research. Furthermore, the study did not include a structured assessment of end-user perspectives regarding cache content or configuration. Future efforts could benefit from incorporating systematic feedback from deployed EMT personnel to further align kits with evolving field needs.

Second, cache maintenance and storage demand systematic planning, along with sustained financial and human resources. Replacing worn-out equipment remains a challenge, as is often the case in low- and middle-income countries (LMICs), particularly in PICs. Additionally, securing warehouse space with adequate environmental controls is difficult in many Pacific contexts, further complicating long-term cache sustainability.

Third, food for team members was excluded from the cache kits. While military-style “meals ready to eat” offer flexibility and self-sufficiency, they are costly and require strict rotation due to their limited shelf life, leading to potential waste. Instead, Pacific EMTs rely on local markets for food procurement at the time of deployment, a “just-in-time” approach that reduces storage needs but increases risk. Disrupted supply chains could make food access unreliable in a major catastrophe, delaying deployments and straining operations.

Fourth, Pacific EMTs typically lack dedicated transport and must rely on external partners, including police, fire departments, the military or private sector assets, adding a layer of uncertainty to response efforts. This dependency makes transport planning a key aspect of preparedness. Deployment logistics must align with cache weight, volume and team size to ensure rapid and efficient mobilization.

Fifth, operational preparedness in disaster response remains an underexplored area, with limited literature guiding best practices. Strengthening health emergency logistics in the Pacific requires a deeper focus on procurement, pre-deployment quality assurance, emergency transportation, communications, warehousing, infrastructure and post-deployment maintenance. Addressing these gaps will be key to improving the speed and effectiveness of future EMT operations.

In conclusion, designing, developing and procuring cache kits for national EMTs in the Pacific provides a scalable model for other EMTs and their partners in LMICs. However, ensuring long-term impact requires further action. Sustaining cache readiness depends on ongoing training, maintenance and operational planning. Additionally, documenting and sharing EMT cache logistics experiences is essential for improving disaster response efficiency and strengthening future operations. Prioritizing these efforts will help build a more resilient emergency response system – not just in the Pacific, but worldwide.
